# Clinico-pathological initial outcome of a newly adopted novel surgical technique for nodal metastatic thyroid cancer at a large-volume centre in a high-income developing country

**DOI:** 10.3389/fsurg.2023.1204230

**Published:** 2023-06-12

**Authors:** Iyad Hassan, Lina Hassan, Farooq Bacha, Mohammad Alsalameh, Omran Qatee, Wiam Hassan

**Affiliations:** ^1^ Department of Surgery, Burjeel Hospital, Abu Dhabi, United Arab Emirates; ^2^ Department of Endocrinology, Burjeel Hospital, Abu Dhabi, United Arab Emirates

**Keywords:** advanced thyroid cancer, minimally invasive neck dissection, vocal cord palsy, intraoperative neuromonitoring, cervical lymph node metastasis

## Abstract

**Introduction:**

Thyroid cancer is the most common tumour in the endocrine system. In the past decade, the incidence of lymph node metastasis has increased, and so has the patient demand for a smaller scar. This research reports the surgical and patho-oncological short-term outcomes of a novel, minimally invasive neck dissection approach for thyroid carcinoma with lymph node metastases at the UAE's leading endocrine surgery centre.

**Methods:**

This study employed a prospectively maintained surgical database to retrospectively analyse relevant parameters such as surgical complications, including bleeding, hypocalcaemia nerve injury and lymphatic fistula, as well as oncological metrics such as the type of tumour and the ratio of lymph node metastasis to the number of harvested lymph nodes in 100 patients that underwent open minimally invasive selective neck dissection.

**Results:**

The study comprised 50 patients with thyroidectomy, with bilateral central compartment neck dissection (BCCND; 50%); 34 with thyroidectomy, BCCND and selective bilateral lateral compartment neck dissection (BLCND; 34%); and 16 patients with selective unilateral central and lateral compartment neck dissection by recurrent nodal disease (ULCND; 16%). The female-to-male gender ratio was 78:22, with median ages of 36 and 42 years, respectively. Histopathology findings revealed that 92% of patients had papillary thyroid cancer (PTC) and 8% had medullary thyroid cancer. The mean total number of lymph nodes removed was 22 in the BLCND group, 17 in the ULCND group and 8 in the BCCND group (*p* = 0.001). Furthermore, the average lymph node metastasis was significantly higher in the BLCND group (*p* = 0.002). The percentage of temporary hypoparathyroidism was 29.8% and it persisted for 1.3%. The lateral compartment dissection morbidity was as follows: Four male patients with tall cell infiltrative PTC presented with pre-existing vocal cord paresis, necessitating nerve resection and anastomosis, and two more developed it after surgery (1.1% of the nerve at risk). Lymphatic fistulas occurred in four patients (4%) who were treated conservatively. Two patients were readmitted due to symptomatic neck collection. Horner syndrome was discovered in just one female patient. Male gender, aggressive histology and lateral compartment dissection independently increased surgical morbidity. In a high-volume endocrine centre, the adoption of minimally invasive selective neck dissections to treat nodal metastatic thyroid cancer did not increase specific cervical surgery complications.

## 1. Introduction

Among different malignant endocrine tumours, thyroid carcinoma is one of the most common; it accounts for almost 1% of all types of malignant tumours and one-third of head-neck malignant tumours (1, 2). In the past decade, however, aggressive thyroid cancer with nodal diseases, which is more prevalent in females than males—about 2–3 times more common—has grown (3). Thus, thyroid surgery, including neck dissection, has become more common globally. In the UAE, the occurrence of thyroid cancer has increased alarmingly compared to neighbouring and Western countries, and it is the second most common malignancy in females (4). Thyroid carcinoma has four main histologic types: differentiated papillary, follicular, medullary and anaplastic (aggressive undifferentiated tumour) (5). Papillary thyroid carcinoma (PTC) is the most common type of thyroid cancer, with 80%–85% prevalence (2). Its incidence is also increasing at an alarming rate (6). The metastasis of lymph nodes in PTC generally occurs in the central compartment, which then expands to the lateral compartment of the neck. However, in some cases, the disease progression may skip metastasis (7, 8). By contrast, medullary malignancy is a heterogenous disease that occurs bilaterally, with an unpredictable nature of multiple endocrine neoplasia, e.g., type IIa are presumably mostly affected by this type of tumour, and it cannot be treated effectively via chemotherapy or radiation (9, 10). Therefore, surgery in that region is the most suitable treatment method (9). A well-planned surgical treatment is therefore necessary to reduce the chances of recurrence, as well as the development of postoperative complications and the formation of scars (1, 11).

Minimally invasive surgeries yield better cosmetic results. These newer techniques were introduced carefully due to technological challenges, new complications and scepticism regarding the oncologic effectiveness (Rossi et al. 2021). The original treatment provided by professionals reduced the likelihood of disease recurrence and the risk of complications (12). Optimising the initial treatment introduces a trade-off between a less aggressive surgery that is associated with recurrent disease and a more aggressive surgery, which may result in postoperative complications (12). In either case, a neck dissection surgery is not without complications. The most common complications include hypoparathyroidism in the central neck dissection and injury to the recurrent laryngeal nerve in both the central and lateral neck dissections (12, 13). Both can be permanent or transient, depending on the surgical technique's traumatic effects (13). The development of a scar on the neck after surgery is another concern that can have a substantial influence on a patient's quality of life, particularly for young women. Therefore, it is crucial to assess the risk factors carefully.

Standard therapy for thyroid cancer with lymph node metastases is total thyroidectomy with lymph node dissection, which typically requires a lengthy vertical component, despite the 6–8 cm long Kocher incision in the suprasternal notch (14). Therefore, a small incision is preferred by many surgeons and patients alike. Recent developments in surgical technology and the patient aesthetic criteria have led to alternative thyroid gland and cervical lymph node metastatic access. Several innovative surgical approaches for neck dissection have also been developed. These include video-assisted thyroidectomy, transoral thyroidectomy and robotic thyroidectomy (15–17). However, they are not without their disadvantages. Robotic thyroidectomy cannot retrieve many central compartment lymph nodes compared to conventional open thyroidectomy (18). Minimally invasive cervical surgeries provide better cosmetic results for the patients and they are also associated with fewer postoperative complications (9, 19). Currently, patients' expectations about their appearance are significantly influenced by social media and celebrities. Additionally, a rising number of surgeons are using social media for advertising purposes in response to increasing competition. These two aspects, in conjunction with the ongoing refinement of surgical instruments, are the primary drivers behind the growth of novel, minimally invasive treatments. Generally, a low, transverse incision is best for postoperative scarring because it runs parallel to and does not penetrate the relaxed skin-tension line. With a low transverse incision, it may be harder to access the upper neck, especially level II, than with other incisions because the incision site is farther away. However, modern energy devices, such as the ultrasonic scalpel, have helped surgeons to overcome this barrier and have allowed them to dissect the upper neck relatively easily without the need for a large incision. Though, there have been concerns that this approach could lead to insufficient lymph node excision and worse oncological outcomes (11). Therefore, in this study, minimally invasive selective lymph node dissection with a low Kocher incision measuring 6 cm was performed in three different ways—bilateral central compartment neck dissection (BCCND), modified bilateral lateral compartment neck dissection (BLCND) and unilateral lateral compartment neck dissection (ULCND). Finally, postoperative complications and clinicopathological parameters were analysed and compared.

## 2. Materials and methods

A prospectively maintained surgical database for patients with thyroid cancer undergoing neck surgery has been established at an endocrine surgery centre in the United Arab Emirates. Data from 100 consecutive thyroid cancer patients who had bilateral or unilateral selective neck dissection by a single surgeon (I.H.), in a tertiary institution between January 2015 and August 2022, were retrospectively analysed. Patients with clinical evidence of lymph node metastasis from thyroid cancer that was confirmed by a preoperative biopsy, who had undergone therapeutic central or centro-lateral compartment dissection with simultaneous thyroidectomy, whose final histopathologic results showed lymph node metastases, and who had at least 30 days of follow-up were included in our study. Patients with thyroid lymphoma, distant metastases or cervical lymph node metastases that could not be cleared were not included in the study. Standard operating room (OR) procedures for thyroid surgery at our institution, including patient placement on the OR table, the selection of anaesthesia, equipment setup and intraoperative neuromonitoring, have been previously described in detail (20). In addition to standard patient characteristics, laboratory results and imaging studies, this prospective database compiles pre-, intra- and postoperative clinicopathological parameters that are particularly relevant to the development of surgical morbidity in patients with thyroid cancer. Along with the usual non-procedure-specific parameters such as wound infection, bleeding, seroma and cardiopulmonary complications, ENT-performed video laryngoscopy before and after surgery, the quantity and quality of drains, a cervical neurovascular examination, and parathormone and calcium measurements at first postoperative day (pod1), tenth (pod10), and 90th (pod90) are the benchmarks by which the quality of our surgery was evaluated. Every acquired pathology specimen at our institution was subjected to microscopic evaluation, and the results of that examination were analysed and double-checked by at least one other qualified pathologist. In this research, the pathological aspects that were investigated included the size and kind of thyroid cancer, the number of cancer foci that were identified across the whole thyroid and the presence of macroscopic tumour invasion into advanced tissues such as the vocal cord nerve and the carotid sheet structures. Furthermore, microscopic invasion markers include lympho-vascular and capsular invasion, the number of excised lymph nodes, the number of metastatic lymph nodes and the ratio of metastatic lymph nodes to harvested lymph nodes were also investigated.

### 2.1. Surgical technique

**Figure 1:**
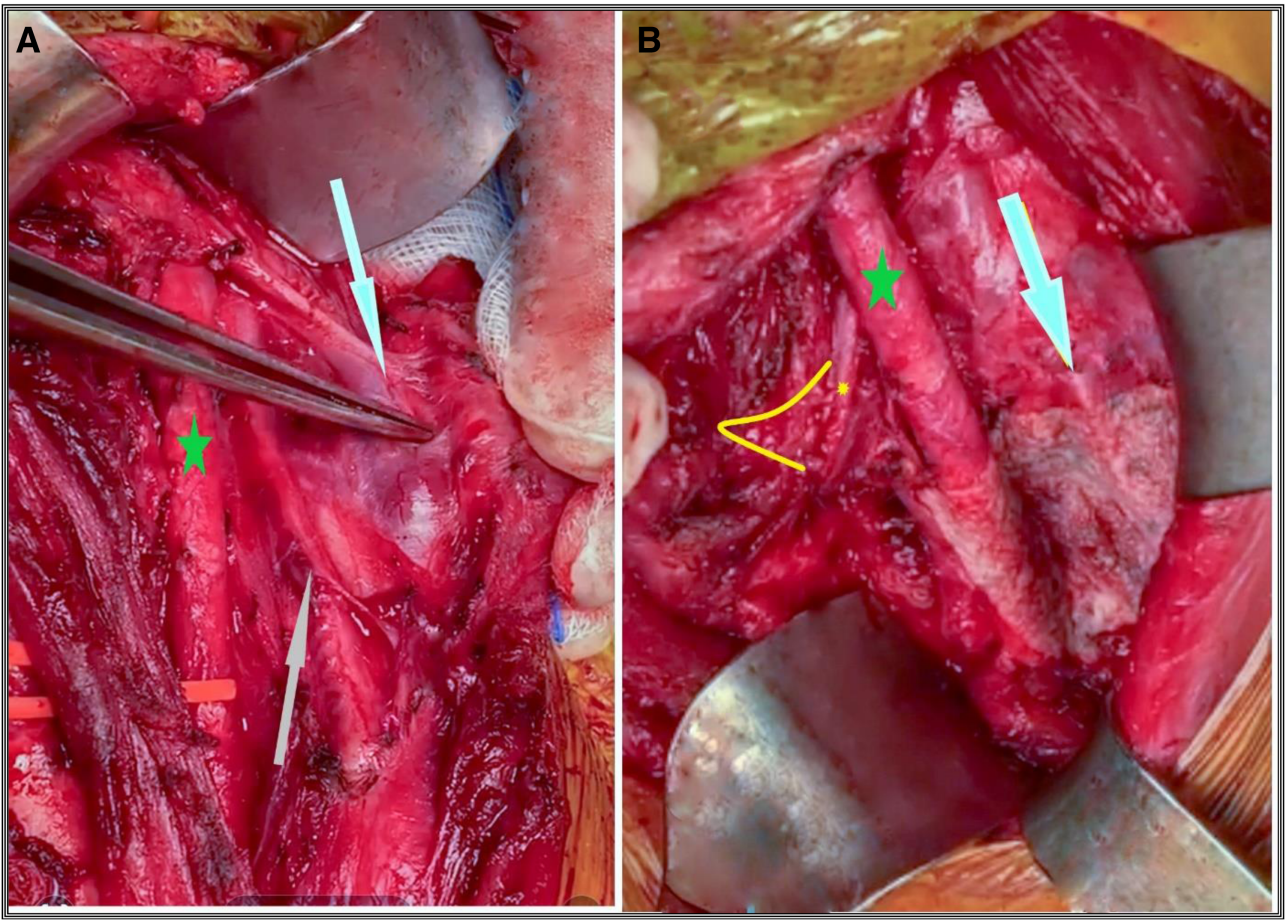
(**A**) The lateral lymph node of medullary thyroid cancer at levels III and IVa invading the internal jugular vein (white arrow) and the vagal nerve (grey arrow); the green star is the common carotid artery. (**B**) Status after completion of left LCND. White arrow indicates the phrenic nerve; small yellow star and arc show the vagal nerve anastomosis to the recurrent laryngeal nerve at the level of its entrance into the larynx after complete block resection of LCND, and the green star is the common carotid artery.

The size and placement of the incision for a total thyroidectomy with BCCND may vary depending on factors such as tumor size and location, as well as individual patient factors. As a general guideline, the incision typically measures between 2.5 and 4 cm in length and is placed 2 cm above the suprasternal notch.” The incision was made 1–1.5 cm longer on either side across the medial margin of the sternocleidomastoid muscle if BLCND or ULCND was also performed (Figures 1A,B). In cases where prior surgery had been performed, the original scar was repurposed. After positioning the patient on the OT table, the ultrasound marking of all suspicious lymph nodes was performed to optimise the location of the transverse incision and to allow for adequate lymph node clearance. The incision was performed as low as possible, and in a skin crease if one was available; otherwise, it was positioned higher but parallel to the skin-tension line if the patient had clear positive lymph nodes at level II, with the aim of optimising aesthetic results. Aside from complete thyroidectomy and BCCND, we routinely dissected levels II through IV of the neck bilaterally or unilaterally on the side of the tumour, if a preoperative FNAC confirmed that lymph nodes were malignant after the prior clinical and imaging examinations were suspicious.

“The specimens from each side's lymph node clearance dissection (LCND) were sent separately for definitive histopathology, whereas the specimen from the bilateral central neck dissection (BCCND) was sent as a single sample”. Series of loops were inserted around the jugular vein, carotid artery, strap muscles and sternal head of the sternocleidomastoid muscles to allow for safe manipulation and lymph node excision. However, the manipulation of the vagus nerve via looping or otherwise was avoided unless absolutely necessary, to minimise the risk of vocal cord paresis originating from the vagal nerve. The hypoglossal nerve served as the dissection's upper border, while the clavicle marked its lower edge. The phrenic nerve marked the posterior border of the LCND. To reduce the risk of chylous fistula, the thoracic duct was always covered with tissel® adhesive or hemopatch® (Baxter, Advanced Surgery Inc, CA, USA). Auto-transplantation of the parathyroid glands was conducted in patients whose lower parathyroid glands were at risk of devascularisation during CCND. During the surgery, the recurrent laryngeal nerve (RLN) was identified using anatomical landmarks such as the middle thyroid artery, the ligament of Berry, and the tracheoesophageal groove”. “Towards the end of the operation, the RLN and the Vagal nerve (VN) were neuro-monitored proximally (VN at the level of the carotid bifurcation) to detect any paresis in the vocal cords that might have been induced by VN damage”. Only in cases of complex lateral lymph node dissection a 12-French suction drain was temporarily placed. For stitching the platysma and subcutaneous tissue layers of the upper and lower skin flaps, absorbable polylactic acid® (4.0 VicrylTM; Ethicon, Inc., cincinciati, OH, USA) was used. Absorbable monofilament (5.0 Biosyn; Ethicon, Inc, cincinciati, OH, USA) was used to close the skin.

### 2.2. Statistical analysis

The student's *t*-test was used to compare the means of continuous variables. When the sample size was small, we used the chi-squared test to compare continuous variables, and Fisher's exact test for categorical variables. Non-parametric variables were distinguished between groups using the Mann-Whitney test. The statistical test ANOVA was used to assess if there are any statistically significant differences among the means of the three groups. SPSS 22.0 was used for all statistical testing (SPSS® Chicago, IL, USA). To draw conclusions from the data, a *p* value of less than 0.05 was considered as statistically significant.

## 3. Results

Lymph node dissection was performed on 100 patients in the following ways: 50 bilateral central compartment neck dissection (BCCND) (50%), 34 BCCND with selective bilateral lateral compartment neck dissection (BLCND) (34%), and 16 patients with recurrent disease received unilateral CCND with selective unilateral lateral compartment neck dissection (ULCND) (16%). The preoperative data of all the patients are summarised in Table 1.

**Table 1:** Direct comparison of preoperative data, stratified by the extent of neck dissection.

Preoperative Data			
	BLCND	ULCND	BCCND
Number of Patients (*n*)	34	16	50
Female/Male (ratio)	8/26	5/11	9/41
Age (years)	37	42**	35
Tg (µg/l)	94.9	28.4	45.1
TgAb (IU/l)	76.13	332.54*	269.47
TPO (IU/l)	107.9	78.2	137.1
Preoperative RAI (*n*)	0	11 (69%)	0
Preoperative RLN Paresis (*n*)	3	1	0

* Denotes a statistically significant difference between groups of patients undergoing BLCND, ULCND and BCCND; *t-test, *p* = 0.008.

** Mann-Whitney test, *p* = 0.019.

Patients who underwent unilateral lateral compartment resection were significantly older than the patients in the BCCND group (Mann-Whitney test, *p* = 0.019). In all three groups, the number of male patients was lower than the female patients. The thyroglobulin protein (Tg) level in patients undergoing BLCND was higher than the other two groups, indicative of a higher cancer load (21, 22). On the other hand, patients undergoing BLCND had a significantly higher amount of mean antithyroglobulin antibody (*t*-test, *p* = 0.013). TgAb is an accurate indicator of assessing the recurrence of thyroid carcinoma in patients in postoperative situations (23). Three patients undergoing BLCND and one undergoing ULCND had preoperative recurrent laryngeal nerve (RLN) paralysis. The four patients were all men. In two patients, the Vagal nerve (VN) was resected due to lymphatic invasion, and the VN was subsequently anastomosed to the recurrent laryngeal nerve (RLN).” (Figure 1A), while the remaining two patients had RLN segment resection and anastomosis. Radioactive iodine (RAI) was performed in 11/16 (69%) patients of the ULCND group. The three surgical approaches to neck dissection were distinct from one another in key aspects. Their data can be found in Table 2. Compared to the other two techniques, the BCCND operation lasted for significantly less time, as anticipated. Two patients undergoing BLCND required the resection of the internal jugular vein due to metastatic invasion (Figure 1). The current practice also preserves the internal jugular vein whenever possible, because sacrificing it may sometimes cause fatal complications (24). Vagal nerve to RLN nerve resection was necessary for two patients in all the cases. The anastomosis of the vagal nerve to RLN (Figure 1B), and RLN to RLN, was also the same. PTC microcarcinoma and the follicular variant of PTC were not significantly different among the three groups. In comparison to the other two forms of dissection, the BLCND procedure significantly yielded the highest average number of lymph nodes removed, and the greatest number of metastases.

**Table 2:** Direct comparison of surgical and pathological parameters, stratified by the extent of neck dissection.

Intraoperative Data			
	BLCND	ULCND	BCCND
Surgery Duration (in minutes)	184* (*p* = 0.001)	137	85
Internal Jugular Vein Resection (*n*)	1	1	0
Vagal Nerve/RLN Resection and Anastomosis (*n*)	3	1	0
Unilateral/Bilateral Foci (*n*)	19/15	5/11	31/19
Medullary Cancer (*n*)	8	0	0
Classic Papillary Cancer (*n*)	16	7	33
Follicular Variant of PTC (*n*)	6	4	7
PTC Microcarcinoma (*n*)	4	5	10
Average Number of LN Removed (*n*)	25* (*p* = 0.001)	17	8
Average Number of Metastasis (*n*)	10 (*p* = 0.002)	8	2
Average Size of Tumour (mm)	24	19	17
Focality Ratio uf/mf (ratio)	20/14* (*p* = 0.026)	13/3	39/11

* Denotes a statistically significant difference between groups using the ANOVA means comparison test.

While the laterality of the multifocal tumours did not change amongst the three techniques, the average size of the tumour excised was greater in the BLCND group (24 mm), compared to the ULCND and BCCND groups (19 mm and 17 mm, respectively).

The PTH level on the first postoperative day (pod1) did not differ significantly between the two primary procedures, BCCND and BLCND, but both were significantly less than the ULCND procedure. Temporary hypoparathyroidism occurred in a maximum of 13 patients on whom the BLCND method was used, and 15 with the BCCND method, but this was not observed when the ULCND procedure was used. The fact that postoperative RLN paralysis was less severe than before surgery is further proof of the efficacy of our surgical technique. Operations carried out with the BLCND method were completely successful, with no need for re-surgery, while the other methods required additional surgery: at least twice with the ULCND method and four times with the BCCND method. Readmission, chylous fistula and hematoma/seroma occurred at most in two patients when the BLCND method was used, none when the BCCND method was used, and only one patient had chylous fistula when the ULCND method was used, proving the effectiveness of our minimally invasive surgery. When the BLCND method was used, Horner syndrome was observed in one patient and no RAI was recorded in patients post-surgery, despite it being present in two patients before the operation. However, while Horner syndrome was not recorded when the ULCND and BCCND methods were used, RAI was recorded in two patients with the ULCND method and three with the BCCND method. The average hospital stay among all the methods was not significantly different. The postoperative data of all the patients are reported in Table 3.

**Table 3:** Direct comparison of the morbidity rates, stratified by the extent of neck dissection.

Postoperative Complication			
	BLCND	ULCND	BCCND
Parathormone Level (pmol/L) pod1	1.79	2.8^†^ (*p* = 0.025)	1.63
Calcium Level (mmol/L) pod1	2.22	2.24	2.21
Temporary Symptomatic Hypoparathyroidism (*n*)	13	0* (*p* = 0.007)	15
Temporary RLN Paresis (*n*)	1	1	0
Readmission (*n*)	2	0	0
Lymphatic Fistula (*n*)	2	1	0
Hematoma/Seroma (*n*)	2	0	0
Horner Syndrome (*n*)	1	0	0
Average Hospital Stay (days)	4	3	2

* Denotes a statistically significant difference between the groups, using a chi-square test.

^†^ Denotes a statistically significant difference between the groups, using the ANOVA means comparison test. (*n*) is the absolute number of cases. pod1 = first postoperative day.

**Figure 2:**
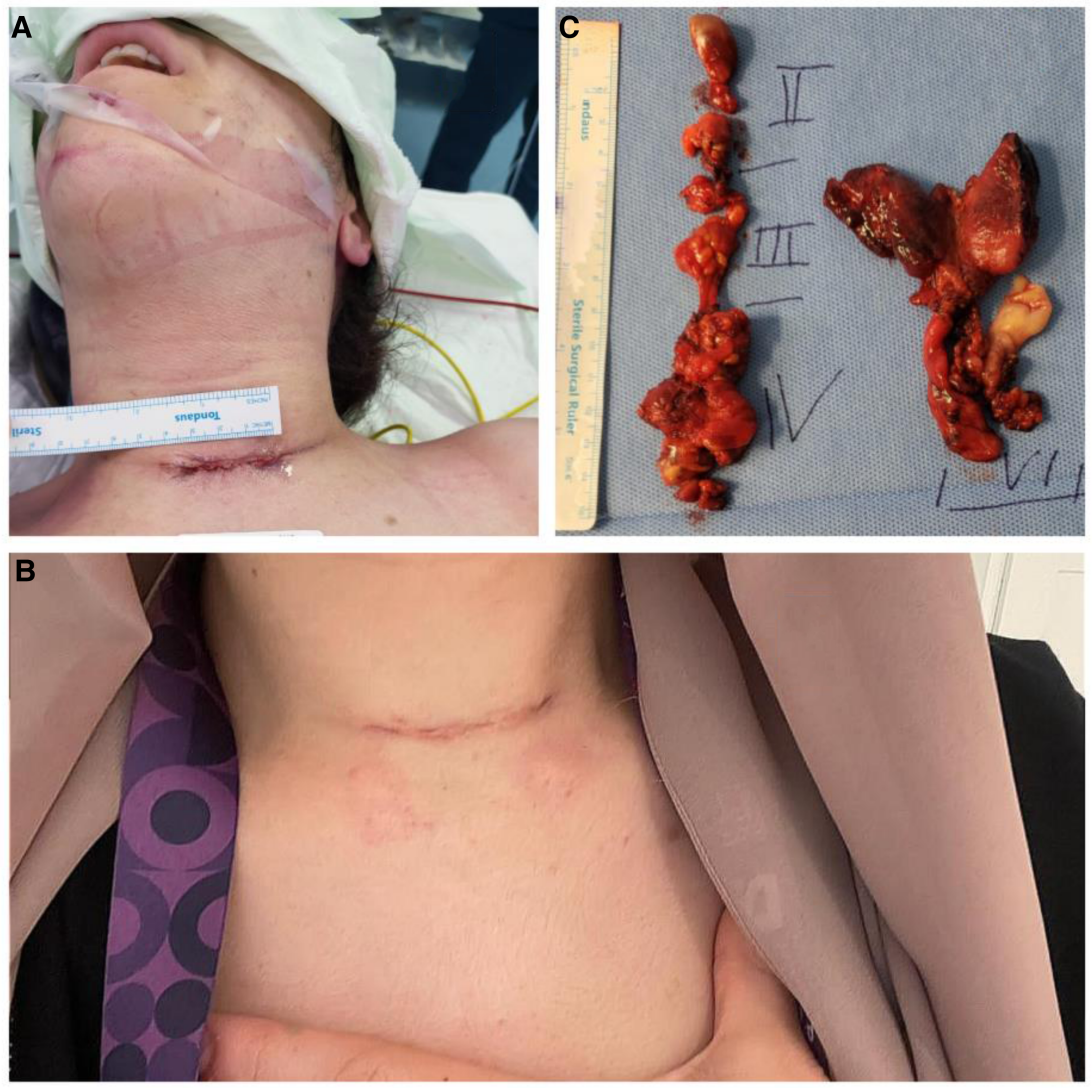
(**A**) 4 cm incision suture of a 31-year-old female patient with multifocal papillary microcarcinoma with clinically nodal diseases. (**B**) Healing skin incision 4 weeks after total thyroidectomy, BCCND and right sided ULCND (**C**) Surgical specimen with final Histopathology which revealed a total of 44 yield LN and a metastatic ratio of 31/44.

Since our minimally invasive surgical method involved using a small incision, and the location of surgery was carefully chosen, scar formation was minimal. Figure 2 demonstrates a patient after ULCND. It is apparent from the image that the patient did not experience any visually unappealing scar formation.

## 4. Discussion

Thyroid cancer therapy has historically been challenging for several reasons, including the potential for undesirable cosmetic effects and complications following surgery (25, 26). Both complications can be reduced by following a minimally invasive surgical method. A thyroidectomy can be considered as minimally invasive if the incision length is between 3.5 and 5.5 cm at its maximum (27). With increasingly stringent cosmetic requirements, a minimally invasive surgery with a smaller incision length is the better option (28). An endoscope-assisted thyroidectomy is performed with a small incision on the neck that results in cosmetic satisfaction for patients (29, 30). However, because of its high cost, many still prefer an open thyroidectomy despite its major disadvantage of leaving a scar afterwards, among other complications (14). A long incision surgery can be the cause of severe postoperative complications such as myofascial pain and adhesive capsulitis because of the obvious formation of scars, which adds to the mental and physical trauma already experienced by the patients (31). With recent advances, smaller incisions are possible, and this technique can reduce the occurrence of such complications and the formation of large scars after surgery (31–33). “In this study”, selective neck dissection was used instead of radical neck dissection in order to reduce post-operative complications and improve patient outcomes by saving the sternocleidomastoid muscle, the vena jugularis interna and the accessory nerve. As opposite to incision used in this study, the traditional “L”-shaped incision usually made from the mastoid tip to a transverse skin fold in the lower neck located just above the sternal notch where patients experienced dissatisfaction with their aesthetic appearance, as well as a chronic tightness sensation and the impairment of neck mobility due to the contracted, tense scar, caused by the crossing of the skin crease lines with subsequent scar contraction, which resulted in visible scars, particularly on the vertical portion (14). Since improving the patient's quality of life is constantly a major priority, we used a minimally invasive technique with a low Kocher incision approximately 6 cm long that is parallel to the normal tension lines of the skin. This allowed us to achieve our surgical goals of removing the tumour while also providing the patient with the best functional and cosmetic recovery possible (Figure 2). Our method involved a selective neck dissection with a short transverse incision, and despite this, we were able to acquire a favourable oncological result with an equal quantity of yielding lymph nodes, comparable to other studies (34). While other authors reported similar findings using a longer incision, our methods yielded, on average, around 25 nodes in the case of BLCND, for example, with a small incision (34). A successful surgery should minimise postoperative complications as much as possible because they can have a negative impact on recovery. In our series, the rate of the well-known surgical complications (such as bleeding, reoperation, paresis of the vocal cords, hypoparathyroidism and readmission) is comparable to, and even lower than, the rate of those complications following a total thyroidectomy alone (34). The ADNM strategy for intraoperative neuromonitoring (20) has been shown to decrease RLN injury during thyroidectomy. Hypocalcaemia and hypoparathyroidism, which can be caused by damaging the blood supply to the parathyroid glands, can be avoided with the help of an endocrine loupe and careful dissection (31). Therefore, in this study, parathormone and calcium levels were measured to gauge the quality of our surgical procedure. These levels were not significantly different among the two primary techniques, and they were also lower compared to other studies where a long incision was made (34). Nevertheless, more sophisticated interventions, such as neurovascular resection with subsequent neural anastomosis, can be conducted safely during the minimally invasive selective neck dissection. Furthermore, the rate of procedure-specific complications, such as lymphatic fistula, was comparable to the international standard. Sealing the thoracic duct using tissel® adhesive and hemopatch® (Baxter®, USA) helped to reduce the number of cases of chylous fistula to only three individuals. However, one case was discovered intraoperatively, and despite clips and 6.0 proline stitches, followed by tissel® glue and a hemopatch®, a minor secretion was still observed after returning to a normal diet on the first postoperative day. Nonetheless, all three patients were treated conservatively; with wound compression, a fat-free diet and 100 mcg of Octreotide injected subcutaneously every 8 h; and it was resolved within a week. Neck dissection risks include postoperative IJV thrombosis. IJV thrombosis has been reported at a rate of 0–29.6% (35). In our study, two patients experienced IJV thrombosis immediately following surgery, which was resolved by their 3-month Doppler scan. The use of energy devices during IJV tissue dissection may have contributed to the incidence of IJV thrombosis. The most commonly injured motoric nerves during lateral neck dissection for head and neck cancers are the spinal accessory nerve, greater auricular nerve and marginal mandibular nerve; phrenic nerve injury is uncommon (36). In our study, none of these nerves showed substantial functional impairment.

This is due to the fact that we adopted a meticulous, nearly protocol-driven, vigilant and proactive minimally invasive surgical dissection technique, paying specific attention to heat or traction near the neural structures. However, one patient with an aggressive tumour with large desmoplastic lymph node metastases exhibited postoperative Horner syndrome. Horner syndrome, a rare neurological complication of thyroid surgery, causes cosmetic concerns but is not life-threatening. It occurs between 0.56% and 9.8% after neck dissection (37). Therefore, surgeons must be cautious while dissecting at level IV, and provide preoperative counselling to patients.

Minimally invasive surgery has been shown to be effective in terms of cosmetic outcomes, speed of recovery, patient satisfaction, and quality of life. However, as a surgical community, we need to make sure that the growing popularity of scarless techniques doesn't put undue pressure on patients to achieve unattainable aesthetic standards promoted by social media and celebrity sources and doesn't exploit patients in an unethical way. However, premature implementation of such procedures at broad adoption has the potential to impede future surgical innovations. Nevertheless, to name just a few of these advancements, there is a possibility of using an endoscopic, robotic, transoral, or hybrid technique for LND, which, in theory, facilitates thorough dissection and merges the advantages of the endoscopic chest approach and the transoral approach. However, not all patients with thyroid cancer qualify as suitable candidates for such strategies because it depends on factors including tumour size, location, and the extent to which lymph nodes are involved, as well as the skill of the surgeon. Nonetheless, once the safety of those approaches is confirmed in a larger cohort, it may be the best surgical procedure for certain individuals (38–41).

Our study had some limitations due to the fact that it was retrospective and without sample size calculation. In spite of the fact that the data were compiled in a prospective database for the purposes of research and quality control, we anticipate that some variables that have the potential to influence the outcome were not recorded in the study. Another restriction is the absence of long-term follow-up, which makes it impossible to determine whether the treatment is superior to the gold standard in terms of oncological and functional outcomes, as well as the quality of life.

## 5. Conclusions

The implementation of minimally invasive selective neck dissections for the treatment of nodal metastatic thyroid cancer in a high-volume endocrine surgery centre is easy and did not result in an increase in the number of particular cervical surgery complications. Larger randomised controlled trials with a long-term follow-up are required to standardise this technique and demonstrate that it is equivalent to the standard neck dissection procedure, particularly in terms of oncological outcomes. These trials are also required to determine if the presumed better functional and aesthetic outcomes could be achieved.

## Data Availability

The raw data supporting the conclusions of this article will be made available by the authors, without undue reservation.
